# Single-Cell Trajectory Inference Guided Enhancement of Thyroid Maturation *In Vitro* Using TGF-Beta Inhibition

**DOI:** 10.3389/fendo.2021.657195

**Published:** 2021-05-31

**Authors:** Mírian Romitti, Sema Elif Eski, Barbara Faria Fonseca, Pierre Gillotay, Sumeet Pal Singh, Sabine Costagliola

**Affiliations:** Institut de Recherche Interdisciplinaire en Biologie Humaine et Moléculaire (IRIBHM), Université Libre de Bruxelles (ULB), Brussels, Belgium

**Keywords:** thyroid, organoid, single-cell, RNA-Seq, TGF-beta

## Abstract

The thyroid gland regulates metabolism and growth *via* secretion of thyroid hormones by thyroid follicular cells (TFCs). Loss of TFCs, by cellular dysfunction, autoimmune destruction or surgical resection, underlies hypothyroidism. Recovery of thyroid hormone levels by transplantation of mature TFCs derived from stem cells *in vitro* holds great therapeutic promise. However, the utilization of *in vitro* derived tissue for regenerative medicine is restricted by the efficiency of differentiation protocols to generate mature organoids. Here, to improve the differentiation efficiency for thyroid organoids, we utilized single-cell RNA-Seq to chart the molecular steps undertaken by individual cells during the *in vitro* transformation of mouse embryonic stem cells to TFCs. Our single-cell atlas of mouse organoid systematically and comprehensively identifies, for the first time, the cell types generated during production of thyroid organoids. Using pseudotime analysis, we identify TGF-beta as a negative regulator of thyroid maturation *in vitro*. Using pharmacological inhibition of TGF-beta pathway, we improve the level of thyroid maturation, in particular the induction of *Nis* expression. This in turn, leads to an enhancement of iodide organification *in vitro*, suggesting functional improvement of the thyroid organoid. Our study highlights the potential of single-cell molecular characterization in understanding and improving thyroid maturation and paves the way for identification of therapeutic targets against thyroid disorders.

## Introduction

### Efficient Generation of Functional Organs *In Vitro* Is Prerequisite for Cell Replacement Therapy

Replacement of damaged or dysfunctional organs by *in vitro* generated tissues is a promising avenue for regenerative medicine. Cell replacement therapy has provided encouraging results against multiple degenerative diseases, including retinal degeneration (1), diabetes (2) and Parkinson’s disease (3). The progress has been enabled by the tremendous advances in development and directed differentiation of pluripotent stem cells - embryonic stem cells (ESCs) and induced pluripotent stem cells (iPSCs). Notably, engineering of pluripotent stem cells to lack components of HLA (human leukocyte antigen), factors that are recognized by the immune system, provides a universal donor material for potential treatment of auto-immune disorders, such as Type 1 diabetes (4, 5). The vast potential of cell replacement therapy is, however, hindered by the ability to mimic organ physiology *in vitro*. Production of patterned organs containing mature, functional cells at high efficiency and purity remains a major bottleneck.

*In vitro* generation of organs with natural architecture and physiology, called organoids, depends on recapitulation of regulatory steps involved in cell fate decisions. To improve our understanding of the process, recently, single-cell RNA-Sequencing (scRNA-Seq) of *in vivo* and *in vitro* organ development has allowed unbiased evaluation of the differentiation process (6–8). scRNA-Seq, by profiling the gene expression levels in individual cells, enables segregation of a heterogeneous cell-population undergoing asynchronous differentiation, thereby allowing identification of signaling pathways active at specific stages of differentiation. Modulation of the identified signaling pathways, particularly those that correlate with terminal differentiation and maturation, can improve the *in vitro* protocol for generation of functional tissues.

Here, we combine scRNA-Seq with an organoid model of the thyroid gland to discern the signaling pathways regulating thyroid differentiation and utilize the information to improve the protocol by modulation of an identified pathway. For this, we exploit a method our group had previously developed for *in vitro* generation of functional thyroid (9). Our thyroid organoid protocol generates 3D, patterned tissue from mouse ES cells. The graft of *in vitro* differentiated thyrocytes was shown to be capable of rescuing a mouse model of complete thyroid loss, underscoring the functional activity of the *in vitro* generated organoid. Though variations of the protocols have been since published (10, 11), two significant shortcomings exist in the field: 1. we lack a comprehensive understanding of the distinct cell lineages present in the organoids, particularly non-thyroid cell types; and 2. we lack systematic analysis of gene regulatory networks underlying the *in vitro* differentiation process. We address these gaps by analysis of mouse thyroid organoid differentiation at single-cell resolution. We comprehensively identify the cell types present in the organoid, decipher the pathways regulating thyroid maturation and finally, utilize this information to improve the efficiency of *in vitro* differentiation protocol.

### Brief Introduction of Thyroid Function and *In Vivo* Development

Thyroid gland is an endocrine organ responsible for synthesis, storage and secretion of thyroid hormones (triiodothyronine (T3) and thyroxine (T4)). The functional unit of the gland are hollow, spherical follicles that store immature thyroid hormone in a colloidal form within the follicle lumen. The follicles are composed of polarized epithelial thyroid follicular cells (TFCs), also known as thyrocytes. Thyrocytes express Thyroglobulin (*Tg*), which serves as the precursor to thyroid hormone. Production of thyroid hormones from *Tg* requires a complex series of reactions that involves bidirectional transport to and from the lumen (recently reviewed in (12, 13)). This process is orchestrated by contributions from multiple genes, including thyroperoxidase (*Tpo*) and sodium/iodide symporter (*Nis*/*Slc5a5*).

*In vivo* the gland develops from the anterior foregut endoderm. The process involves recruitment of a group of endodermal cells to thyroid fate, in an event called “specification” or “determination”, which at the molecular level, is characterized by the co-expression of *Nkx2-1* (TTF-1), *Foxe1* (TTF-2), *Pax8* and *Hhex* transcription factors (14–19). Subsequently, thyroid progenitors undergo a series of events which comprise cell proliferation, invasion of the surrounding mesenchyme, migration, thyroid lobes enlargement and consequent appearing of follicular organization (20, 21). The thyroid differentiation program is complete when the gland reaches its final location and TFCs express a series of specific proteins which are essential for thyroid hormonogenesis. The thyroid markers present a temporal pattern of expression, in which thyroglobulin (*Tg*), thyroperoxidase (*Tpo*), and TSH receptor (*Tshr*) are detected initially (18), followed by sodium/iodide symporter (*Nis*) (22) and *Duox1/2* expression (23), resulting in thyroid hormone synthesis, storage and secretion.

*In vitro* differentiation of thyroid gland from pluripotent stem cells is capable of generating polarized, spherical follicles that synthesize thyroid hormone (9, 11). Here, using single-cell RNA-Seq of mouse thyroid organoid model, we reconstruct the gene expression dynamics during *in vitro* differentiation and utilize it to shed light on the molecular dynamics during thyroid maturation process.

## Materials and Methods

### ESC Culture for Maintenance and Differentiation

**Figure 1:**
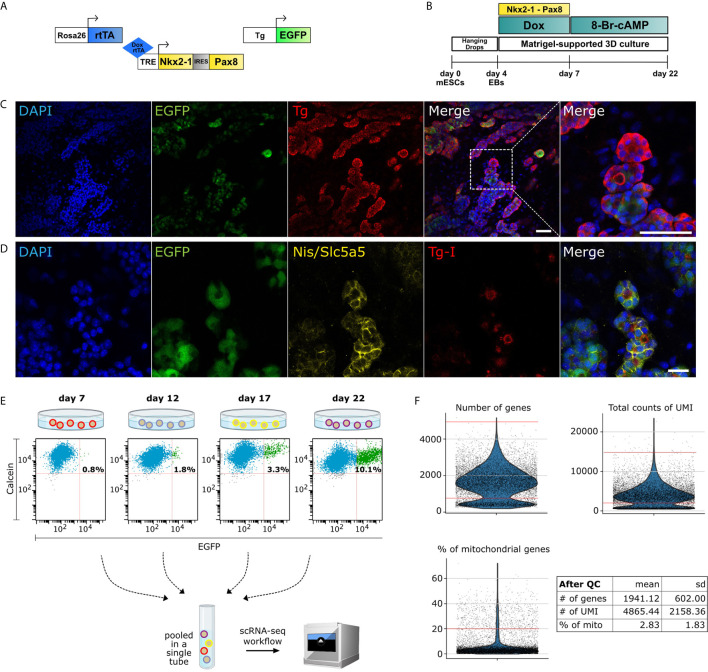
Single-cell RNA-Seq of mouse organoid. **(A)** Schematic of the three constructs used in the protocol: Rosa26 locus driven reverse tetracycline-controlled transactivator (rtTA), Tet-Responsive Element (TRE) driven *Nkx2-1* and *Pax8*, and *Tg* promoter driven EGFP. **(B)** Schematic of differentiation protocol for mESC-derived *in vitro* thyroid organoid. **(C)** Immunofluorescence images from cells at day 22 displaying differentiated organoids marked with DAPI (blue), EGFP (green) and Thyroglobulin (*Tg*) (red). Zoomed image shows spheric thyroid follicles (Scale bars: 50 µm). **(D)** In addition, thyroid follicles express functional markers as Slc5a5/Nis (yellow) and Tg-I (red) (Scale bar: 20 µm). **(E)** Cells from differentiated organoids at days 7, 12, 17 and 22 were collected on the same day and labeled with calcein (live cell marker) and FACS-sorted for scRNA-seq analysis. Percentage of EGFP+ thyrocytes are noted. For downstream analysis, EGFP+ and EGFP- cells (1:1 ratio) from day 12, 17 and 22 (at day 7 no GFP+ enrichment was performed) were pooled into a single tube and sequenced using 10X Genomics platform. **(F)** Violin plots showing the quality control parameters for cells profiled using scRNA-seq. Number of genes, total counts of UMI and the percentage of mitochondrial genes were utilized for quality control. Red lines on violin plots depict the cutoffs for filtered cells. After filtration, cells from scRNA-seq had a mean ± standard deviation of 1941 ± 602 genes, 4865 ± 2158 UMIs and 2.83 ± 1.83% of mitochondrial gene content.

Here, we utilized mouse ESCs that had been engineered to express three constructs (**Figure 1A**): 1. Rosa26 locus driven Tet-On transcription factor (tat: reverse tetracycline-controlled transactivator), 2. At the Hypoxanthine phosphoribosyltransferase (HPRT) locus was inserted Tet-Responsive Element (TRE) driven bicistronic construct containing Nkx2-1 and Pax8, and 3. Bovine *Tg* promoter driven EGFP. The three constructs allow doxycycline-induced expression of exogenous *Nkx2-1* and *Pax8* and monitoring of the thyroid lineage by Tg-driven EGFP expression.

A2Lox.Cre_TRE-Nkx2-1/Pax8_Tg-EGFP mouse ESCs (**Figure 1B**) were cultured and were differentiated as described previously by Antonica, 2012 (9). Briefly, embryoid bodies, generated by hanging drops culturing of ESCs (1,000 cells per drop) were collected after 4 days and embedded in growth- factor-restricted Matrigel (MTG; BD Biosciences); 50μl MTG drops (containing around 20 EBs) were plated into 12-well plates. Embryoid bodies were differentiated using a differentiation medium initially supplemented with 1μg/ml of Doxycycline (Sigma) for 3 days, followed by two weeks of maturation by using differentiation medium containing 0.3 nmol of 8-Br-cAMP (BioLog) (**Figure 1B**). Cells were collected and fixed (PFA 4%) at day 22 in order to confirm the thyroid differentiation (**Figures 1C, D**).

### FACS Sorting of Mouse Thyroid Organoid

At differentiation days: 7, 12, 17 and 22, organoids cultured in MTG drops were washed twice with Hanks’s balanced salt solution (HBSS, containing calcium and magnesium; Invitrogen) and incubated for 30 min at 37°C in a solution (1 ml per well) containing 10 U/ml of dispase II (Roche) and 125 U/ml of collagenase type IV (Sigma) in HBSS. Cells were dissociated and re-suspended manually by using a P1000 pipette and collected in a 15-ml Falcon tube (3 wells per tube, in duplicates, per time point). Then the enzymes were inactivated by adding 10% FBS and cells were centrifuged at 500g for 3 min. Cells were rinsed twice with HBSS following centrifugation settings described above. Finally, the organoids were incubated with TrypLE Select solution (Thermo Fisher, 12563011) for 10 min at 37°C, in order to dissociate into single cells. Following dissociation, TrypLE solution was inactivated with 10% FBS, and single cells pelleted by centrifugation at 500 g for 3 min. The pellet was resuspended in 500 μl of HBSS, and the solution was filtered using a 30 μm cell filter (pluriSelect, 43-50030-03). In order to remove dead cells, calcein violet (Thermo Fisher, C34858) was added (final concentration of 1 μM) to the cell suspension and it was incubated at room temperature for 20 min. The single-cell preparation was sorted with the appropriate gates, including excitation with 405 nm laser for identification of alive cells (calcein+) and excitation with 488 nm for thyroid (Tg-EGFP+) cells (**Figure 1E**). FACS was performed through 100 μm nozzle.

### Single-Cell RNA-Sequencing of the Mouse Thyroid Organoid

For single-cell RNA-seq of the thyroid *in vitro* organoids using the 10× Genomics platform, cell suspension was prepared as mentioned above from the thyroid organoids collected at differentiation days 7, 12, 17 and 22. For each stage, cells were dissociated and collected by FACS as described above. 4000 alive cells for each stage were collected in a single tube. For day 7, cells were collected irrespective of EGFP expression, while for days 12, 17 and 22, 50% EGFP+ and 50% EGFP- cells were collected. Sequencing library from the collected cells was prepared and sequenced following the previously described protocol (24).

In order to build the reference for Cell Ranger, mouse genome (GRCm38) and gene annotation (Ensembl 87) were downloaded from Ensembl and the annotation was filtered with the “mkgtf “ command of Cell Ranger (options: “–attribute = gene_biotype:protein_coding– attri- bute = gene_biotype:lincRNA –attribute = gene_biotype:antisense”). Within, the mouse genome and filtered gene annotation, the last exon of *Pax8* gene was masked. The last exon for *Pax8* was introduced and labeled as exogenous Nkx2-1_Pax8 sequence (10x Chromium 3’ RNA Sequencing kit only sequences ~100 bp upstream of the polyA tail. For endogenous *Pax8*, this would correspond to sequences in the 3’ UTR. For exogenous *Pax8*, this would correspond to sequences in the last exon, as the exogenous *Pax8* does not harbor a 3’ UTR after the coding sequence. The exogenously provided sequence is a bicistronic construct containing *Nkx2-1* and *Pax8*. Hence, levels of exogenous *Pax8* reflect levels of the bicistronic *Nkx2-1*, *Pax8* construct). The modified genome sequence and annotation were then used as input to the “mkref “ command of Cell Ranger to build the appropriate Cell Ranger Reference.

### Cluster Analysis of ScRNA-Seq Data

The raw data generated from Cell Ranger pipeline was processed using Scanpy 1.6.0 package (25) by following the recommended pipeline. Briefly, filtering was performed on raw data to remove low quality cells by filtering cell profiles that had high mitochondrial gene content, low number of captured unique molecules or expressed low number of genes (**Figure 1F**). To remove doublets, cell profiles with high UMI or genes were further excluded (**Figure 1F**). After quality control, raw data was log-normalized, cell cycle score was calculated. The effect of cell cycle, library size and mitochondrial counts was regressed out. Finally, the data was scaled for downstream analysis. Highly variable genes were utilized to compute principal component analysis and neighborhood graph (UMAP). For clustering, the first forty principal components were utilized as they covered 90% of the standard deviation as accessed by elbow plot. Further, a resolution of 0.4 was used for leiden clustering. Differential gene expression analysis was performed for each cluster using Wilcoxon rank-sum test to identify marker genes.

To subcluster the thyroid lineage, distributions of normalized gene expressions were plotted for *Tg, Slc5a5, Tpo, Hhex*, *Foxe1, Nkx2-1* and *Pax8.* For each gene, cells expressing the gene above 0.5 times the average normalized expression were labeled as expressing cells.

### Gene Ontology (GO) Analysis

GO term analysis was performed using Enrichr (26) by using the “crisp gene set” input method by submitting the complete differentially expressed gene list obtained using Wilcoxon rank-sum test. Genes that were statistically enriched (False Discovery Rate (FDR) < 0.05) with a minimum log fold enrichment of 1.5 within a cluster were chosen for analysis. For visualization, statistically significant (p-value < 0.05) terms were selected from GO Biological Process 2018 (top four or five most significant; KEGG 2019 Human, WikiPathways 2019 Human, and GO Molecular Function 2018 (most cell-type related relevant terms.

### Diffusion Pseudotime Analysis of ScRNA-Seq Data

For single cell trajectory inference, diffusion pseudotime was computed following the standard analysis pipeline (26) provided by Scanpy package. Since a single lineage was selected for the analysis, number of branchings was set to 1 and Dox-responsive cells were selected as root cells. To visualize the marker genes trajectory on trendline plot, plot.wishbone_marker_trajectory function used was provided by Wishbone package (27).

### Pharmacological Inhibition of TGF-Beta Pathway

In order to inhibit TGF-beta signaling, thyroid organoids were cultured in differentiation medium containing cAMP and SB431542 inhibitor (10μM; Peprotech) from day 22 to day 29 of differentiation protocol and the medium was changed every 48 h. At day 29, thyroid organoids were collected for gene expression, immunofluorescence and iodide organification.

### Quantitative PCR (qPCR) Analysis

qPCR was performed on cDNA generated from thyroid organoids at differentiation day 29. For this, total RNA was extracted from thyroid organoids by lysis using RLT Lysis buffer (Qiagen) + 1% 2-mercaptoethanol. Extracted RNA was isolated using RNeasy micro kit (Qiagen) according to the manufacturer’s instructions. Reverse transcription was done using Superscript II kit (Invitrogen) to generate cDNA. qPCR was performed on cDNA in triplicates using Takyon (Eurogentec) and CFX Connect Real-Time System (Biorad). Results are presented as linearized values normalized to the housekeeping gene, *B2microglobulin* and the indicated reference value (2-DDCt). The gene expression profile was obtained from three independent samples. Primers used were as follows: *B2microglobulin*: Fw 5’- GCTTCAGTCGTCAGCATGG-3’, Rv 5’-CAGTTCAGTATGTTCGGCTTCC-3’; *Nkx2-1*: Fw 5’-GGCGCCATGTCTTGTTCT-3’, Rv 5’-GGGCTCAAGCGCATCTCA-3’; *Pax8*: Fw 5’-CAGCCTGCTGAGTTCTCCAT-3’, Rv 5’-CTGTCTCAGGCCAAGTC CTC-3’; *Foxe1*: Fw 5’-GGCGGCATCTACAAGTTCAT-3’, Rv 5’- GGATCTTGAGGAAGCAGTCG-3’; *Tshr*: Fw 5’-GTCTGCCCAATATT TCCAGGATCTA-3’, Rv 5’-GCTCTGTCAAGGCATCAGGGT-3’; *Slc5a5 (Nis)*: Fw 5’-AGCTGCCAACACTTCCAGAG-3’, Rv 5’-GATGAGAGCAC CACAAAGCA-3’; *Tg*: Fw 5’-GTCCAATGCCAAAATGATGGTC-3’, Rv 5’-GAGAGCATCGGTGCTGTTAAT-3’; *Tpo*: Fw 5’-ACAGTCACAGTTCT CCACGGATG-3’, Rv 5’-ATCTCTATTGTTGCACGCCCC-3’; *Duox2*: Fw 5’ -AACGGCACTCTCTGACATGG-3’, Rv 5’ -GGCCCCATTACCTTTTTGCC-3’.

### Immunofluorescence

For protein immuno-detection experiments, cells were fixed in 4% paraformaldehyde (Sigma) for 1 h at room temperature (RT) and washed three times in PBS. Cells were blocked in a solution of PBS containing 3% bovine serum albumin (BSA; Sigma), 5% horse serum (Invitrogen) and 0.3% Triton X-100 (Sigma) for 30 min at RT. The primary and secondary antibodies were diluted in a solution of PBS containing 3% BSA, 1% horse serum and 0.1% Triton X-100. Primary antibodies against Tg (rabbit anti-TG, A0251 Dako, 1:2,000), Nis (rabbit anti-NIS, a gift from N. Carrasco, 1:1,000), Tg-I (mouse anti-TG-I, a gift from C. Ris-Stalpers, 1:100), beta-III tubulin (mouse anti-Tubb3 (TUJ1), MMS-435P-200, Eurogentec, 1:1,000), alpha-smooth muscle actin (rabbit anti-alpha smooth muscle actin (E184), ab32575, Abcam, 1:1,000) and troponin T (mouse anti-cardiac troponin T, ab8295, Abcam, 1:1,000) were incubated overnight at 4°C followed by incubation with secondary antibodies (donkey anti-mouse and anti-rabbit IgG conjugated with DyLight-Cy3 and DyLight-647; 1:500; Jackson Immunoresearch) and Hoechst 33342 (1:1,000; Invitrogen) for 2 h at RT. Coverslips were mounted with Glycergel (Dako). The samples were imaged on Zeiss LSM 780 confocal microscope using a 32x magnification.

### Iodide Organification Assay

Matrigel-embedded thyroid organoids at differentiation day 29, treated with cAMP or cAMP+SB431542, were initially washed with HBSS and incubated with 1 ml of an organification medium containing 1,000,000 c.p.m. per ml ^125^I (PerkinElmer) and 100 nM sodium iodide (Sigma) in HBSS for 2 h at 37°C. After incubation, 1 ml of 4 mM methimazole (MMI), was added to the cells and washed with ice-cold PBS. In order to remove and dissociate the organoids embedded in Matrigel-droplet, cells were detached using 0.1% trypsin (Invitrogen) and 1 mM EDTA (Invitrogen) in PBS for 15 min. For iodide uptake quantification, cells were collected in polyester tubes and radioactivity was measured with a c-counter. Subsequently, proteins were precipitated twice by addition of 1 mg of gamma-globulins (Sigma) and 2 ml 20% TCA followed by centrifugation at 2,000 r.p.m. for 10 min, at 4°C and the radioactivity of protein-bound [^125^I] (PBI) was measured. Iodide organification was calculated as an iodide uptake/PBI ratio and the values expressed as a percentage. As control for iodide uptake and protein-binding measurements cells were also treated with 30μM sodium perchlorate (Nis inhibitor; NaClO4, Sigma-Aldrich)) and 2 μM methimazole (TPO inhibitor; MMI, Sigma-Aldrich), respectively.

### Statistical Analysis

The experiments were performed using three experimental replicates for qPCR analysis and five replicates for organification assay. Gene expression levels are shown in fold changes and compared to the control condition using Mann-Withney test. Uptake and protein-bound levels are expressed as mean ± SD and compared by unpaired Student’s t-test. 

## Results

### Single-Cell RNA-Seq of Mouse Organoid

Our previously published protocol was used to generate mouse thyroid organoid from mouse embryonic stem cells (mESCs) (9). Here, we utilized mESCs in which *Nkx2-1* and *Pax8* genes, required for thyroid lineage differentiation, are temporally induced with a Tet-On based system (see Methods). The Tet-On system is activated by treatment with doxycycline, leading to expression of exogenous *Nkx2-1* and *Pax8* (referred as *exNkx2-1_Pax8* henceforth). Moreover, this mESC line contains an EGFP transgene under control of bovine Tg promoter, allowing monitoring of differentiation of thyrocytes *in vitro* (**Figure 1A**). It is worth mentioning that in our experience, also corroborated by data presented later, the bovine *Tg* driven EGFP expression *in vitro* is restricted to Tg+ cells but does not recapitulate the entirety of endogenous *Tg* expression. It provides instead a useful indicator of the differentiation status of the culture.

Thyroid organoids are generated from mESCs in a multi-stage protocol (9) (**Figure 1B**). Briefly, the mESCs are differentiated into embryoid bodies (first step towards differentiation, not endoderm/thyroid specific) by day 4, using the hanging drop technique, upon which the cells are embedded in matrigel and treated with doxycycline for three days to induce the expression of exNkx2-1_Pax8 until day 7. At this stage, cells are treated with thyrotropin hormone (or cAMP) until day 22 or longer. In accordance with our previous work, here we demonstrate that thyroid follicular cells are mature by day 22, and express thyroid transcription factors, *Nkx2-1*, *Pax8*, *Hhex* and *Foxe1*, as well as functional markers such as *Tg* (**Figure 1C**), *Tshr*, *Tpo* and *Nis* (**Figure 1D**). Moreover, thyroid cells self-organize into three-dimensional follicular structures containing iodinated thyroglobulin (Tg-I) in the lumen (**Figure 1D**). This indicates the functionality of the model, since it is well known that for hormonogenesis, even at low degrees of iodination, Tg-I forms thyroid hormone (28).

To obtain a cellular resolution understanding of the multi-stage process, we performed single-cell RNA-Seq (scRNA) from cells collected at four stages of the protocol (**Figure 1E**): day 7 (end of doxycycline-based induction of exogenous *Nkx2-1* and *Pax8*), day 12 (early differentiation), day 17 (advanced differentiation), and finally day 22 (functional thyroid follicles). To avoid batch effects, we combined 4,000 cells from each stage into a single tube. For this, four differentiation protocols were initiated sequentially such that the four required stages appear on the day of cell collection. On the same day, organoids from the four stages were dissociated in parallel and sorted by a flow cytometer. Using FACS, 4,000 alive cells were collected from day 7, following which 4000 cells (50% GFP+ and 50% GFP-) were isolated from day 12, 17 and 22 (**Figure 1E** and **Supplementary Figure 1**). Cells from the four stages were mixed in a single-tube to provide 12,000 cells in total, which were profiled using droplet based scRNA-Seq from 10x Genomics. Upon sequencing the library to a depth of 105 million reads and demultiplexing, we obtained profiles from 9,904 cells. The profiled cells underwent quality control on the basis of Unique Molecular Identifier (UMI) (29), number of genes and percentage of mitochondrial reads (**Figure 1F**), yielding a total of 7,381 filtered cells for downstream analysis.

### Identification of Cell Types Present in Thyroid Organoid

**Figure 2:**
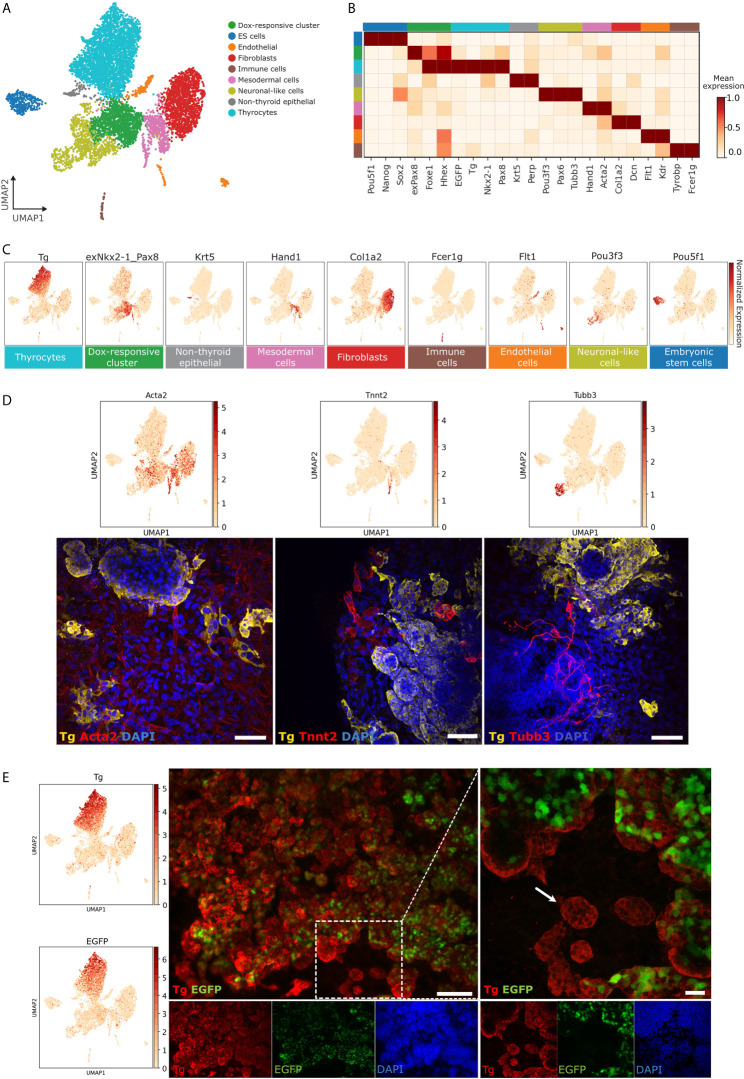
Characterization of cell types present in thyroid organoids. **(A)** Unsupervised clustering of *in vitro* thyroid organoid model. Each cluster is represented by a specific color. **(B)** Gene-expression matrix plot for selected marker genes for each cell cluster. Rows depict cell clusters, while columns depict genes. The intensity of color in each square indicates mean expression within the cluster. **(C)** UMAP expression plots of representative marker genes for thyrocytes (*Tg*), Dox-responsive cluster (exNkx2-1_Pax8), Non-thyroid epithelial cells 1 (*Krt5*), Mesodermal cells (*Hand1*), Fibroblasts (*Col1a2*), Immune cells (*Fcer1g*), Endothelial cells (*Flt1*), Neuronal-like cells (*Pou3f3*) and Embryonic stem cells (*Pou5f1*). Color intensity indicates normalized expression of respective genes. **(D)** Immunofluorescence-based detection of specific non-thyroid cell-types at differentiation day 22. For mesenchymal cell population, fibroblasts were labeled with smooth muscle actin (*Acta2*; red) while cardiomyocytes were marked with Troponin T (*Tnnt2*; red). Thyroid follicles were labeled with antibody against Thyroglobulin (*Tg*; yellow). Neurons were confirmed by staining for beta-III tubulin (*Tubb3*; red). **(E)** To evaluate the extent of bovine *Tg* promoter driven GFP expression in thyroid follicular cells, organoid culture at differentiation day 22 was stained for Tg (red). A fraction of Tg-expressing cells do not express EGFP (Zoomed right figure; arrow shows *Tg*+ EGFP- follicle). Scale bars: 100 µm and 20 µm (zoom).

To visualize distinct cell types in mESC-derived thyroid organoids, we projected the scRNA-seq data onto two dimensions using a non-linear dimensionality reduction technique, UMAP (30), and performed unsupervised graph-based cell clustering (**Figure 2A**). For each cluster, marker genes were identified using Scanpy (25) (**Supplementary Table 1**). Marker genes display statistically significant enrichment in expression levels within a particular cluster. Using the list of marker genes, we annotated cell types with literature surveys. Overall, we identified nine clusters for mESC-derived thyroid organoid model (**Figure 2A**). Of interest, we could identify a cluster of thyroid follicular cells containing 2,914 cells that display expression of genes involved in thyroid gland development and thyroid hormone production, including *Tg*, *Nkx2-1*, *Pax8* as well as EGFP driven from Tg promoter (**Figures 2B, C**).

As described in our previous study where we established the mouse thyroid organoid (9), the protocol generates 25 to 60% cells expressing endogenous *Nkx2-1* and *Pax8* (**Supplementary Figure 2**). The identity and molecular nature of the remaining cells has so far remained undocumented. Using literature survey of marker genes, we annotated the non-thyroid cells present in the organoid model (**Figures 2A–C**). Based on cluster-specific signature genes, we identified 282 embryonic stem cells expressing stemness markers such as *Pou5f1 (Oct4), Nanog*, *Sox2*; 209 endothelial cells enriched in markers such as *Flt1, Kdr, Cd34, Sox17*; 42 immune cells with known markers such as *Tyrobp, Fcer1g, Csf1r, Lyz*; 502 mesodermal cells expressing mesoderm lineage marker genes such as *Hand1, Hand2, Gata6, Acta2*; 1363 fibroblast cells with expression of pan-fibroblast markers such as *Col1a2, Col1a1, Dcn*; 76 non-thyroid epithelial cells with epithelial cell specific marker gene expression such as *Krt5, Krt15*; 910 neuronal-like cells with expression of markers such as *Pax6*, *Tubb3*, *Pou3f3*; and finally 1083 Dox-responsive cells which express a mixture of early thyroid markers and epithelial specific markers in addition to exogenous bicistronic *Nkx2-1/Pax8* construct (exNkx2-1_Pax8) expression.

The presence of specific non-thyroid cell-types identified in scRNAseq analyses (**Figure 2D**) was validated. By immunofluorescence, at differentiation day 22, numerous mesodermal cells were observed, including fibroblasts expressing smooth muscle actin (*Acta2*) and cardiomyocytes expressing Troponin T (*Tnnt2*). Moreover, the presence of neuronal cell population was confirmed by beta-III tubulin (*Tubb3*) staining (**Figure 2D**).

To gain further insights into the gene expression profiles of different clusters, GO Term analysis was performed for marker genes (**Supplementary Figure 3**). This revealed involvement of distinct metabolic and biological processes to each cell type. Of interest, thyrocytes showed an enrichment of genes involved in endocrine system development, thyroid gland development, thyroxine (thyroid hormone) production, NRF2 pathway, TGF-beta and Wnt signaling pathways. Dox-responsive cells were enriched for genes involved in regulation of cell cycle process, regulation of neurogenesis, BMP and Wnt signaling pathways and pluripotency. Neuronal-like cells presented signatures for nervous system development and neuron differentiation. Fibroblast cluster displayed enrichment of genes involved in ECM-receptor expression, collagen fibril organization, matrix metalloproteinases and canonical and non-canonical TGF-beta and Wnt signaling, whereas mesoderm cluster showed enrichment of genes involved in vasculature development, endoderm differentiation and VEGFR signaling pathway.

We also took advantage of our scRNAseq data to better characterize the co-expression of *Tg* and bovine Tg promoter-driven EGFP within thyroid cell population. Within the thyrocyte cluster, *Tg* expression was more prominent than EGFP (**Figure 2E**). As the scRNA-Seq data displayed presence of *Tg*+ EGFP- cells, a careful immunofluorescence analysis of the organoid at day 22 was performed. With this, thyroid follicular cells expressing TG but not EGFP (**Figure 2E**) can be readily observed, thereby underscoring the heterogeneity in EGFP expression within the thyrocyte population.

### Pseudotime Analysis Reveals Gene Expression Dynamics During Thyroid Differentiation

**Figure 3:**
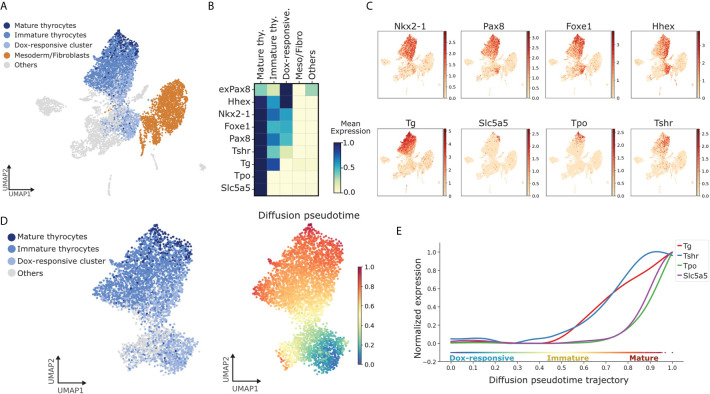
Identification of pathways dynamically regulated during TFCs maturation. **(A)** UMAP displaying subclusters within the thyroid lineage (Dox-responsive cluster, immature and mature thyrocytes). Remaining cells are labeled as ‘Others’. **(B)** Gene-expression matrix plot for thyroid markers: *exPax8*, *Hhex*, *Nkx2-1*, *Foxe1*, *Pax8*, *Tshr*, *Tg*, *Tpo*, *Nis*/*Slc5a5* in subclusters of thyroid lineage and other cell groups. Color intensity in each square represents mean expression in the corresponding cluster. **(C)** UMAP overlaid with gene expression plots for thyrocyte markers. Color indicates normalized expression. **(D)** Diffusion pseudotime analysis of thyrocyte lineage. (Left) UMAP displaying the subclusters within the thyroid lineage; (Right) UMAP overlaid with pseudotime. Color in the pseudotime plot indicates the ordering of the cells. **(E)** Expression trends of thyroid differentiation markers along pseudotime trajectory.

Next, we focused on the thyrocyte cluster to gain detailed information on the factors regulating thyrocyte morphogenesis. For this, we noted that while thyrocytes uniformly expressed *Thyroglobulin* (*Tg*), they did not uniformly express genes related to thyroid functionality (**Figures 3A–C**). Of note, only a small proportion of thyrocytes expressed *Tpo* and *Nis*/*Slc5a5*. Next, we labeled *Tg*+ *Tpo*- *Slc5a5*- cells as immature thyrocytes and *Tg*+ *Tpo*+ or *Tg*+*Slc5a5*+ cells as mature thyrocytes. Mature thyrocytes constituted 7% of all thyrocyte population. It is worth noting that immature thyrocytes express *Nkx2-1*, *Pax8*, *Foxe1* and *Hhex* (**Figures 3B, C**), and while the expression of *Tshr* was low, it was more uniform than the expression of *Tpo* or *Slc5a5* within the thyrocyte cluster (**Figure 3C**).

Further, to extend the analysis to the cellular source of thyrocytes, we included cells labeled as ‘Dox-responsive cells’, as defined by cells that co-express exogenous or endogenous *Nkx2-1* and *Pax8*, along with *Hhex* and *Foxe1*, but lack *Tg* expression (**Figure 3B**). This annotation was based on the expression of known marker genes for thyroid progenitors (14). Remaining cells in the atlas were labeled as ‘Others’.

By combining dox-responsive cells with thyrocytes segregated by maturation status, we could obtain a cellular progression recapitulating thyroid differentiation and maturation. Using the progressive nature of thyrocyte morphogenesis, an *in silico* temporal ordering of the thyrocyte cluster was constructed. In this, a trajectory of cellular progression from one state to another is predicted based on similarity in gene expression (31). Using pseudotime analysis, the transition starting from dox-responsive cells was estimated. It is worth pointing out that the pseudotime analysis indicates two possible differentiation branches originating from Dox-responsive cells (**Figure 3D**). One of the branches moves towards non-thyroid lineages, suggesting that part of the cells expressing exogenous *Nkx2-1*/*Pax8* can differentiate to non-thyrocyte lineages (**Figure 3D**). This potentially explains the presence of non-thyroid cell types in the thyroid organoid model and suggests presence of additional factors that specifically restricts the progenitors to thyroid lineage. The presence of non-thyroid cell types, in part, might be as well explained by the absence of endoderm-induction and anteriorization steps in our protocol. However, the second branch, as expected, moved towards mature thyrocytes (**Figure 3D**). This transition was used to generate the trend of gene expression along the trajectory (**Figure 3E**). Due to the limitation of our model, where thyroid specification is forced by Dox-overexpression of *Nkx2-1* and *Pax8*, we focused our analysis on genes related to thyroid maturation. Interestingly, *Tg* and *Tshr* display similar trends, while the expression of *Slc5a5* and *Tpo* increase steeply at the end of pseudotime trajectory (**Figure 3E**). This demonstrates the reliability of the organoid system in recapitulating the steps of thyroid maturation *in vitro*.

### TGF-Beta and Planar Cell Polarity Display Dynamic Regulation During Thyroid Maturation

**Figure 4:**
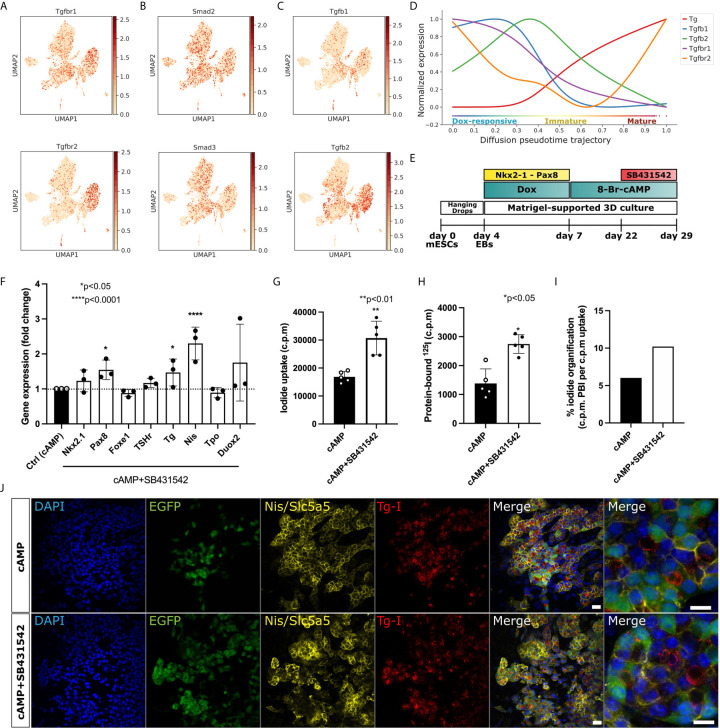
Enhancement of maturation efficiency by pharmacological inhibition of TGF-beta. **(A)** UMAPs demonstrate TGF-beta receptors (*Tgfbr1* and *Tgfbr2*), **(B)** activators (*Smad2* and *Smad3*) and **(C)** ligands (*Tgfb1* and *Tgfb2*) are expressed in mesoderm cells and in thyroid cluster. **(D)** Expression trends of TGF-beta receptors and ligands along pseudotime trajectory in thyroid cells. **(E)** Schematic representing the thyroid differentiation protocol to test the impact of TGF-beta inhibition on thyroid organoid maturation. TGF-beta inhibition was induced by co-treatment with cAMP+SB431542 from day 22 to day 29. **(F)** qPCR analysis demonstrates significant upregulation in *Pax8*, *Tg* and *Nis* gene expression after 7 days of TGF-beta pathway inhibition. *p<0.05, ****p<0.001 by Mann-Whitney test **(G–I)** Functional organification assay confirms significant improvement in 125I uptake **(G)** and protein-bound 125I **(H)** under SB431542 treatment, which results in a higher percentage of cells with capacity of 125I organification **(I)**. **p< 0.01, *p<0.05 by Student’s t-test. **(J)** Immunofluorescence staining demonstrating that SB431542 treatment does not affect thyroid follicular organization evidenced by *Nis* specific expression and Tg-I presence in the lumen space Scale bar: 20 µm and 10 µm (inset).

To identify key signaling pathways regulating the maturation of thyrocytes, trajectory analysis was used to identify genes and pathways that correlate with the progression in thyroid lineage. Further, the gene expression was compared with mesodermal/fibroblast lineage, which acted as a negative control. With this, two pathways which could be pivotal in thyrocyte lineage progression were identified: TGF-beta signaling pathway (**Figures 4A–D**) and Non-canonical Wnt/Planar Cell Polarity (PCP) pathway (**Supplementary Figure 4**). Indeed, cells belonging to thyrocyte, mesoderm and fibroblasts clusters express higher levels of TGF-beta receptors, *Tgfbr1* and *Tgfbr2*, as well as downstream activators, including *Smad2* and *Smad3*. While the expression of the ligands *Tgfb1* and *Tgfb2* are more pronounced in mesoderm/fibroblast cells (**Figures 4A–C**). Moreover, using pseudotime analysis, we estimated the trend in gene expression of TGF-beta receptors and ligands from Dox-responsive cells to mature thyrocytes. Interestingly, we observe that the expression of *Tgfb1*, *Tgfb2* and *Tgfbr1* decreases steeply upon maturation of thyrocytes. In contrast, *Tgfbr2* shows a distinct pattern, which increases during the transition from immature to mature thyrocyte state (**Figure 4D**). Besides TGF-Beta signaling pathway, positive regulators for PCP pathway, such as *Lrp5*, *Porcn*, *Fzd5*, *Dvl1*, *Celsr1* and *Vangl1*, are enriched in thyrocyte lineage and increased during maturation (**Supplementary Figure 4**). In contrast, inhibitors of PCP pathway, such as *Dkk2*, *Sfrp1*, *Sfrp2*, *Wif1*, are exclusively expressed in mesoderm/fibroblasts. Overall, these results suggest that dynamics in TGF-beta pathways and Wnt/PCP may play a decisive role in thyrocyte differentiation and maturation.

### Inhibition of TGF-Beta Pathway Improves the Efficiency of Thyroid Maturation *In Vitro*

In order to assess the scRNAseq-detected potential inhibitory effect of TGF-beta pathway on maturation of thyrocytes, organoids were treated with TGF-beta receptor inhibitor, SB431542 (10 μM), in addition to cAMP, for seven days at the end of the differentiation protocol (From day 22 to day 29; **Figure 4E**). Interestingly, we observed that TGF-beta inhibition results in significant induction of *Tg* and *Pax8* gene expression (p<0.05; **Figure 4F**). In addition, a pronounced effect on *Nis* regulation was observed, since SB431542 treatment resulted in more than 2-fold induction in the *Nis* expression level compared to the control (p < 0.0001; **Figure 4F**). *Nis*/*Slc5a5* transports iodide into thyrocytes. Based on our findings, as well as existing data suggesting an inhibitory effect of TGF-beta pathway on thyroid function (32, 33), we hypothesized that an increase in *Nis* expression due to TGF-beta inhibition could improve the capacity of the thyrocytes to concentrate iodide from the culture media. To test such improvement in thyroid function, we performed iodide organification assay (9). In line with our hypothesis, the inhibition of TGF-beta pathway increased the capacity of thyrocytes to uptake iodide (p < 0.001; **Figure 4G**), while also improving the efficiency of iodide-binding to Tg (p < 0.05; **Figure 4H**). Consequently, this leads to a higher percentage of cells promoting iodide organification (**Figure 4I**). In addition, immunofluorescence staining confirmed that the TGF-beta inhibition did not affect thyroid follicular organization and demonstrated specific *Nis* expression with Tg-I presence in the lumen space (**Figure 4J**).

## Discussion

Substantial progress has been made in uncovering the molecular regulators of thyroid development and function (14, 15); yet less than 5% of congenital thyroid disorder cases have a known genetic cause (34, 35). A better and wider characterization of the key regulators of thyroid differentiation is required to understand the development of thyroid disorders. Here, by taking advantage of unbiased single-cell RNA sequencing of our previously established mESC-derived thyroid organoid model, we have generated a molecular map of the thyroid gland lineage at single-cell resolution. Moreover, we identified some key signaling pathways driving thyroid cell differentiation. Specifically, our analysis suggests the involvement of TGF-β along with the non-canonical Wnt/PCP pathway in regulating thyroid gland lineage. Moreover, pharmacological manipulations of the TGF-β pathway confirmed its inhibitory effect on thyroid maturation and function.

Thyroid-stimulating hormone (TSH) is the main primary physiological regulator of thyroid growth and function (36). Besides TSH/cAMP and inositol phosphate (IP) signaling, recent studies have suggested extrinsic regulators that tightly control folliculogenesis and maturation. Among them, multiple lines of evidence indicate a regulatory role of transforming growth factor-beta 1 (TGF-β1) and epidermal growth factor (EGF) ligands on thyroid cell proliferation, differentiation and function (37, 38). Our observations support previous studies performed in rat and porcine cells, which demonstrated that TGF-beta overexpression affects thyroid growth, gene expression and function. Among the functional markers, the inhibition of *Nis* expression was associated with reduction of iodide uptake and consequently iodide organification (32, 33, 37–39). This *in vitro* model mimics the aspects of *in vivo* thyroid functionality and may help to identify receptors or ligands controlling TGF-beta pathway during thyrocyte differentiation as well as in pathological conditions.

Further, mouse thyroid maturation occurs as a consecutive cascade of events which can be identified by the expression of specific thyroid markers (14). Taking this temporal gene expression into account, we could order individual thyrocytes along a linear differentiation trajectory (**Figure 3D**). Moreover, GO analysis revealed enrichment of markers related to Nrf2 pathway in thyroid cells cluster (**Supplementary Figure 3**). It has been demonstrated that in physiological conditions, Nrf2 coordinates antioxidant defenses, directly regulating thyroglobulin expression and prevents its excessive iodination. Importantly, dysregulation of this pathway has been demonstrated in pathological conditions such as multinodular goiter and thyroid cancer (40). Thyroid organoids constitute a valuable tool to explore molecular mechanisms and could be used to better understand Tg regulation by Nrf2 as well as its role in thyroid development. In addition to the study of TGF-beta and BMP pathway, our trajectory analysis suggests the enrichment of Wnt/PCP signaling components, both ligands and receptors, specifically in mature thyrocytes (**Supplementary Figure 4**). Given the role of Wnt/PCP in the differentiation and function of endocrine cells in the pancreas (41–43), our data warrants a detailed analysis of Wnt/PCP pathway in the polarized thyroid follicular cells.

Besides the molecular characterization of the thyroid organoid, our study provides a map of the cell-types present in the culture. Notably, we observe the presence of fibroblasts in the organoid model. Fibroblasts are part of the connective tissue present in the thyroid gland (24, 44). Fibroblasts play an essential role in the physiology of an organ. For instance, fibroblasts have been shown to be critical for homeostasis of intestinal epithelial *in vivo* and in organoid cultures (45–47). Notably, sub-populations of intestinal fibroblasts express non-canonical Wnt ligands and BMP inhibitors (48), generating the necessary microenvironment for maintenance of stem cell niche. Further, remodeling of collagen by fibroblasts has been implicated in the progression of thyroid cancer (49). Thus, the presence of fibroblasts in the thyroid organoid model provides an opportunity to study the role of stroma in thyroid biology.

The current study provides the first single-cell atlas of mouse thyroid organoid, yet it has certain limitations. Firstly, the number of thyrocytes with mature characteristics (*Tg*+ *Tpo*+ *Slc5a5*+) profiled in the study are rather low, approximately 1.3% of the total profiled cells. In future, it would be of interest to enrich for mature thyrocytes, potentially by exclusively profiling EGFP+ population in late stages of differentiation. Secondly, we lack an *in vivo* scRNA-Seq reference of endocrine differentiation into thyroid follicles. Currently, a single-cell atlas of adult thyroid gland for zebrafish (24) and humans (50), and bulk RNA-Seq data of mouse thyroid progenitors (51) is present in literature. However, we lack a detailed atlas of anterior foregut endoderm undergoing thyroid differentiation at cellular resolution. Without an *in vivo* reference, we are missing information on potential factors that are absent *in vitro* but could potentially improve the maturation of the organoid.

Further, in future, it would be of interest to establish a multi-dimensional atlas of the thyroid organoid and organ by simultaneous profiling of chromatin accessibility and protein levels along with transcriptome, potentially by utilizing protocols that forgo enzymatic dissociation (52) to reduce genetic perturbation associated with cell collection. Such integrative dataset would allow development of gene regulatory networks active during distinct stages of thyroid differentiation. This could elucidate redundancy and feedback loops present in the system, allowing better identification of gene targets for improving the robustness of differentiation protocol.

## Limitation of the Study

Our study provides the first single-cell RNA-Seq evaluation of the thyroid organoid. However, it is restricted by the simultaneous profiling of multiple stages (**Figure 1E**). Here, four time-points were combined into one reaction tube and profiled simultaneously. This helps remove any batch effects introduced by the sequencing reaction. Further, as our goal was to understand the molecular dynamics during thyroid maturation, this experimental setup provided us information about cells at different stages of the differentiation process. However, it removed information related to the time-point of origin of the cell. We invested effort into validating the cell-types using immunofluorescence (**Figure 2D**) to showcase the presence of the cells at the final stage of the organoid model. However, due to the lack of separation of cell-types by differentiation time-point, we are unable to perform detailed analysis of the cell-cell communication by ligand-receptor mapping. In future, it would be of interest to profile each stage individually, possibly even by combining with multi-omics, to gain insight into cell-cell communication and crosstalk occurring in the thyroid organoid system.

## Data Availability Statement

All NGS data (raw files and counts) are uploaded to GEO with accession number GSE163818. Scripts to analyze the data and metadata are made available on Github at https://github.com/selifeski/mouse-thyroid-invitro. The data can also be accessed from an interactive Shiny App at https://sumeet.shinyapps.io/mouse-thyroid-invitro/.

## Author Contributions

SS and MR conceptualized the project. MR and BF generated and maintained thyroid organoids. SS performed the single-cell RNA-Sequencing. SE performed the bioinformatics analysis. PG performed confocal imaging, MR performed pharmacological manipulations and with help of BF performed the subsequent analysis. MR, SE, and BF wrote the first draft and SC and SS edited the manuscript. SC and SS acquired funding for the project. All authors contributed to the article and approved the submitted version.

## Funding

Work by SS was supported by MISU funding from the FNRS (34772792 – SCHISM). This work was supported by grants from the Belgian National Fund for Scientific Research (FNRS) (FRSM 3-4598-12; CDR-J.0145.16, GEQ U.G030.19), the Fonds d’Encouragement à la Recherche de l’Université Libre de Bruxelles (FER-ULB). This project has received funding from the European Union’s Horizon 2020 research and innovation programme under grant agreement No. 825745.

## Conflict of Interest

The authors declare that the research was conducted in the absence of any commercial or financial relationships that could be construed as a potential conflict of interest.

## Supplementary Material

Supplementary Table 1 Differentially expressed marker genes. Marker genes display minimum 1.5 log-fold change and FDR (false discovery rate) of less than 0.05.
