# Supplementary material for: Single-Cell Trajectory Inference Guided Enhancement of Thyroid Maturation In Vitro Using TGF-Beta Inhibition
Source: Front Endocrinol (Lausanne). 2021 May 31;12:657195. doi: 10.3389/fendo.2021.657195 (PMC8202408; doi:10.3389/fendo.2021.657195)
Supplement: Supplementary file 1 [file DataSheet_1.pdf]

Supplementary Figures

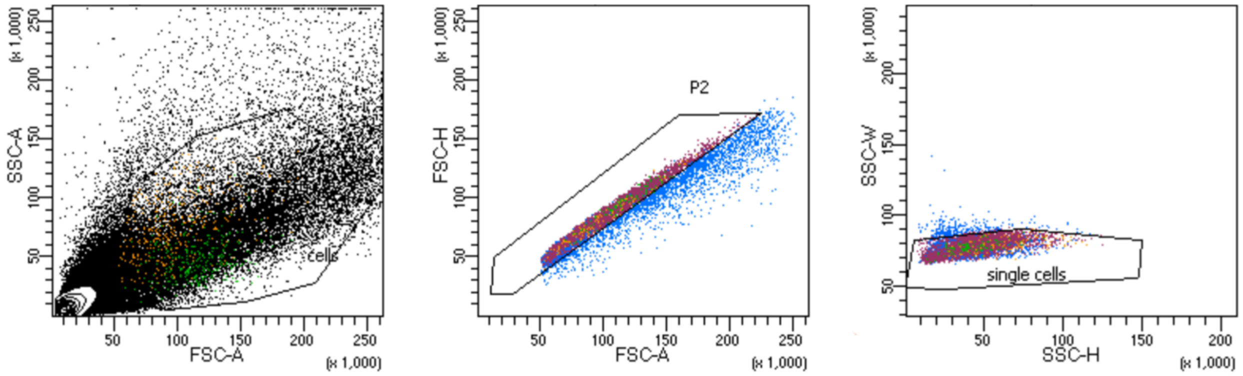

Supplementary Figure 1. Flow cytometry plots showing the gating strategy used for cell sorting. Cells were identified using a FSC-A/SSC-A plot and doublets were excluded based on gates in FSC-A/FSC-H and SSC-H/SSC-W plots. After, single cells were chosen according to calcein violet (viability) and EGFP+ fluorescence, as shown in Figure 1.

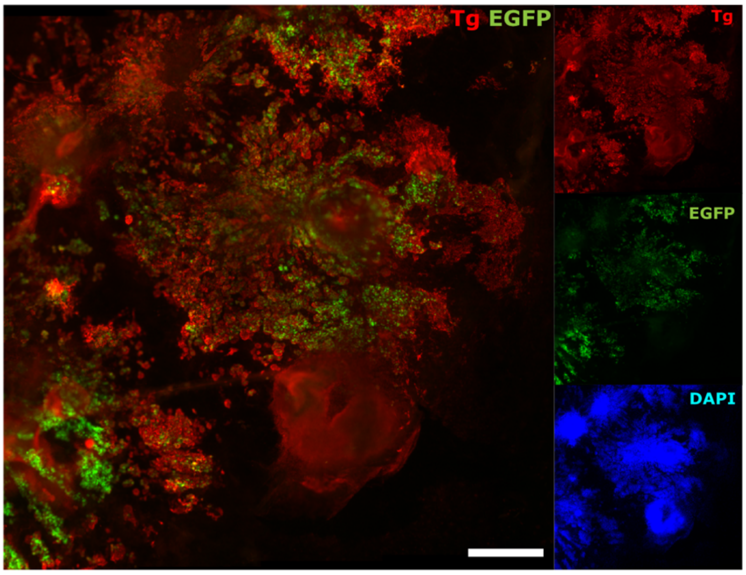

Supplementary Figure 2. Large view of thyroid organoids culture at differentiation day 22 shows several follicles expressing Tg (red) while a smaller proportion expresses the bTg_EGFP reporter (green) (Scale bar: 500 µm).

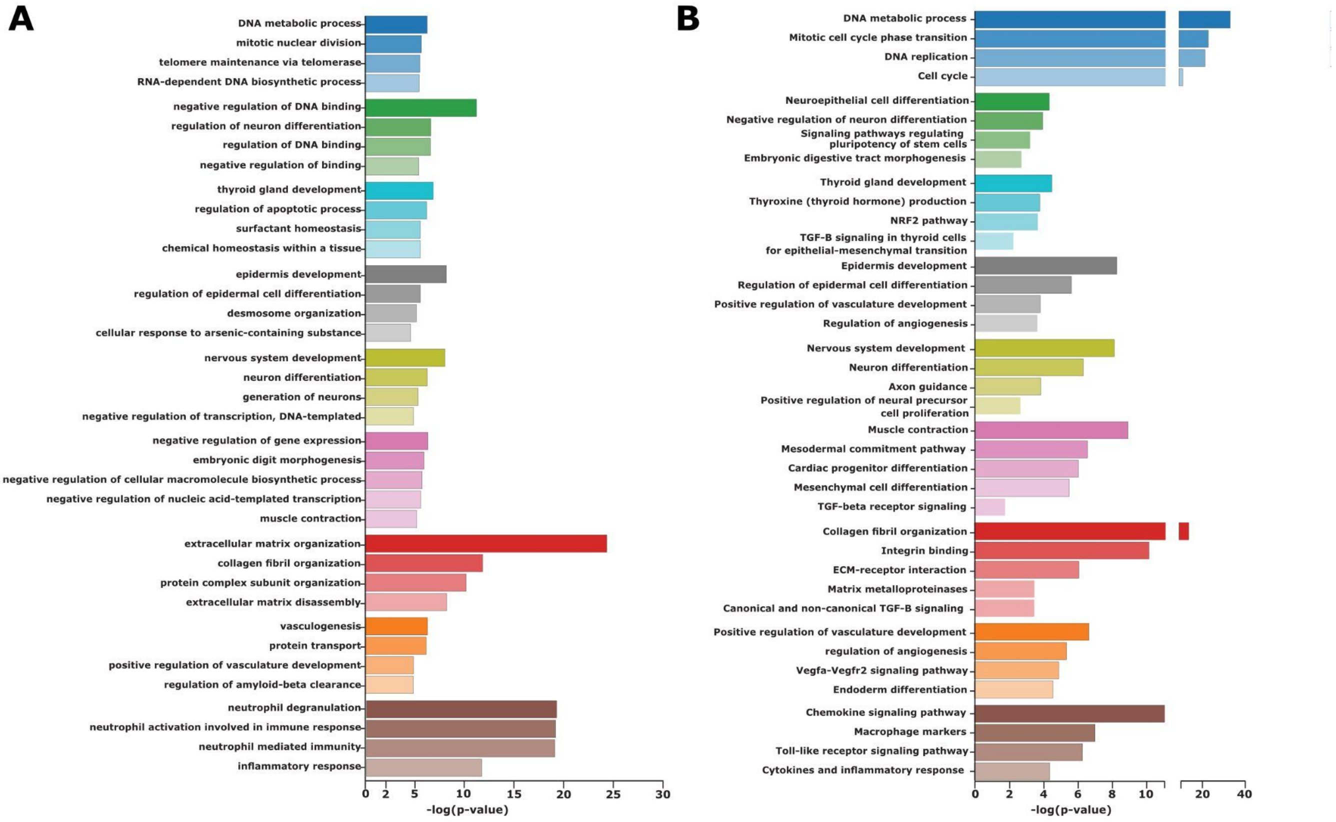

Supplementary Figure 3. GO term analysis for differentially expressed genes among the identified cell clusters. **(A)** The first more significant GO terms for each cluster according to biological processes classification. Color of the bar plots correspond to the color of the respective cell cluster. **(B)** GO terms selected according to the literature and relevance inside each cell type cluster.

- Dox-responsive cluster
- ES cells
- Endothelial
- Fibroblasts
- Immune cells
- Mesodermal cells
- Neuronal-like cells
- Non-thyroid epithelial
- Thyrocytes

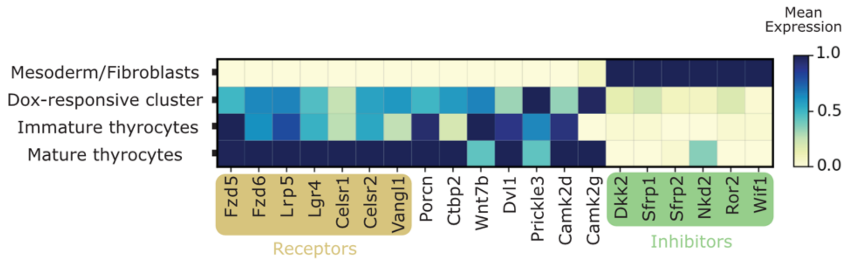

Supplementary Figure 4. Gene-expression matrix plot demonstrates increase in gene expression of Wnt/PCP receptors and effectors in mature thyroid cells compared to immature and Dox-responsive clusters. Mesoderm/fibroblast is used as a non-thyroid cell control.
